# Agreement between the Schedule for the Evaluation of Individual Quality of Life-Direct Weighting (SEIQoL-DW) interview and a paper-administered adaption

**DOI:** 10.1186/s12874-020-00961-9

**Published:** 2020-04-10

**Authors:** Marion Burckhardt, Steffen Fleischer, Almuth Berg

**Affiliations:** 1 Institute of Health and Nursing Science, Medical Faculty, Martin Luther University Halle-Wittenberg, Halle (Saale), Germany; 2 SRH University of Applied Health Sciences, Gera, Germany

**Keywords:** Quality of life, Patient reported outcome measures, Psychometrics, Clinical trials, Cardiac surgery

## Abstract

**Background:**

The Schedule for the Evaluation of Individual Quality of Life-Direct Weighting (SEIQoL-DW) is a prevalent face-to-face interview method for measuring quality of life by integrating respondent-generated dimensions. To apply this method in clinical trials, a paper-administered alternative would be of interest. Therefore, our study aimed to analyze the agreement between the SEIQoL-DW and a paper questionnaire version (SEIQoL-PF/G).

**Methods:**

In a crossover design, both measures were completed in a random sequence. 104 patients at a heart surgery hospital in Germany were randomly assigned to receive either the SEIQoL-DW or the SEIQoL-PF/G as the first measurement in the sequence. Patients were approached on their earliest stable day after surgery. The average time between both measurements was 1 day (mean 1.3; SD 0.8).

Agreement regarding the indices, ratings, and weightings of nominated life areas (cues) was explored using Bland-Altman plots with 95% limits of agreement (LoA). Agreement of the SEIQoL indices was defined as acceptable if the LoA did not exceed a threshold of 10 scale points. Data from *n* = 99 patients were included in the agreement analysis.

**Results:**

Both measures led to similarly nominated cues. The most frequently nominated cues were “physical health” and “family”.

In the Bland-Altman plot, the indices showed a mean of differences of 2 points (95% CI, − 1 to 6). The upper LoA showed a difference of 36 points (95% CI, 30 to 42), and the lower LoA showed a difference of − 31 points (95% CI, − 37 to − 26). Thus, the LoAs and confidence intervals exceeded the predefined threshold. The Bland-Altman plots for the cue levels and cue weights showed similar results.

The SEIQoL-PF/G version showed a tendency for equal weighting of cues, while the weighting procedure of the SEIQoL-DW led to greater variability.

**Conclusions:**

For cardiac surgery patients, use of the current version of the SEIQoL-PF/G as a substitute for the SEIQoL-DW is not recommended.

The current questionnaire weighting method seems to be unable to distinguish weighting for different cues. Therefore, the further design of a weighting method without interviewer support as a paper-administered measure of individual quality of life is desirable.

## Background

Quality of life (QoL) as a patient-reported outcome has growing importance in clinical research [1]. A variety of QoL measures with different dimensions and underlying theoretical assumptions have been developed to assess QoL [2]. Nevertheless, no consensus exists regarding the essential domains of the QoL [3]. Traditional QoL measures often emphasize physical dimensions, although mental and social dimensions should be more prominent from a stakeholder perspective [3]. Attempts to incorporate individually relevant values and dimensions into QoL measurements by using more respondent-generated QoL measures to determine individuals’ QoL have increased [4]. The Schedule for the Evaluation of Individual Quality of Life (SEIQoL) [5–7] is a measure that allows respondent-generated QoL dimensions. This measure is an interview-based assessment of QoL from an individual’s perspective. In contrast to researcher-generated instruments that use predefined QoL dimensions, the SEIQoL offers the opportunity to nominate, weight, and rate dimensions that are considered important for QoL from an individual’s unique perspective. However, this approach requires more time and effort during the data collection phase. Therefore, a short form of the instrument - the SEIQoL-Direct Weighting method (SEIQoL-DW) [8, 9] - was developed and used in numerous studies with a variety of populations [10, 11]. Convergent and discriminant validity of the SEIQoL-DW can be described as moderate-to-high for global QoL, life satisfaction, and mental health but weak for the functional status and health measures [10].

The use of this short form has some pragmatic limitations in research, largely because it must be delivered in a personal semi-structured interview. Wettergren et al. [10] described mean administration times ranging from 5 to up to 50 min for one single SEIQoL-DW interview. This requirement limits the applicability and feasibility of this measure in research, because considerable resources for data collection are required. Therefore, some paper and computer adaptations of the SEIQoL-DW have been developed [12–16]. However, in a preliminary literature search, we were not able to identify any data regarding agreement between the original SEIQoL-DW and an adaption administered in written form.

Therefore, our study aims to explore the agreement between the SEIQoL-DW and a self-developed, paper-administered version of the SEIQoL-DW that assesses the following factors:

1. agreement between the two SEIQoL indices;
2. agreement between the cue levels in each cue; and
3. agreement between the cue weights in each cue.

Our paper-based questionnaire was intended for postal use in a clinical trial and hereafter will be referred to as the Schedule for the Evaluation of Individual Quality of Life-Paper Form/German language (SEIQoL-PF/G). Since the person determines their own QoL concept, patient understanding of the draft instrument was evaluated according to the ISPOR recommendations [17] for content validity using cognitive interviews [18]. The feasibility of the resulting version was tested within a randomized controlled trial with patients in cardiac surgery, general surgery, and internal medicine [19].

## Methods

We describe the methods and results of our study according to the Guidelines for Reporting Reliability and Agreement Studies (GRRAS) checklist [20].

### Study design

The study was designed as a crossover trial in which both measures were completed by the patients in a random sequence as follows:

- Sequence group 1:
  first measurement SEIQoL-DW, second measurement SEIQoL-PF/G,

- Sequence group 2:
  first measurement SEIQoL-PF/G, second measurement SEIQoL-DW.

The rationale for choosing this study design was to control for effects of order of presentation in the completion of the instruments.

### Measures

#### SEIQoL-DW

The SEIQoL-DW is administered in a face-to-face interview. After an introduction of the QoL concept from the individual’s perspective, the SEIQoL-DW is performed in three steps. First, the interviewee chooses the five individual aspects that are the most important areas of their life (cues). If the respondent cannot nominate five cues, a prompt list with examples is provided. The interviewer documents the meaning of each cue as labeled by the respondent (cue label). Second, the interviewee rates their current performance (cue level) in each area on a bar chart. Finally, the interviewee assigns weights to the nominated areas in relation to each other on a special weighting disc in the form of a pie chart (cue weight). During each stage, the interviewers provide comprehensive support through explanations and examples. The outcomes of the SEIQoL-DW are cue labels and their definitions, cue levels and cue weights. The levels are scored using the height of the bar charts (0 to 100), which yields five scores from independent continuous measurements. From the sum of the products of the levels and the relative weights of the five cues, an index can be calculated and used for comparisons on an individual or group level with a range from 0 to 100 [8, 9].

#### SEIQoL-PF/G

The SEIQoL-PF/G (see Additional file 1) is constructed as a paper questionnaire version in the German language and is self-explanatory for self-administration without support from an interviewer. The major modifications of the SEIQoL-DW include the addition of brief written instructions, a prompt list with examples of the most common cues, and an explanation of how to use the visual analogue scale (VAS) in the questionnaire. With the VAS, rating and weighting is conducted for each cue. The index is calculated by adding the products of the levels and the relative weights of the five cues. The relative weight of a cue is calculated by dividing the weight of the cue in the VAS by the sum of the weights of the VASs of all cues [18].

### Setting and participants

The most common application of the SEIQoL-DW is the description of the QoL of patients with specific chronic or incurable diseases [10]. Because the feasibility of the SEIQoL-PF/G was previously tested with cardiac surgery patients [19], the study was conducted in a similar population between May 2013 and June 2013 in the Sana Heart Surgery Hospital in Stuttgart, Germany. The hospital covers the normal spectrum of cardiac surgery types. After surgery, patients are monitored for an average of one to 2 days in the intensive care unit. Once their condition is stable, they are transferred to a general ward. On average, patients are transferred to a rehabilitation facility after eight to ten days.

Patients were eligible for the study if they had completed surgery and, from a clinician’ point of view, they were physically and cognitively able to fill out a questionnaire. Additional inclusion criteria were an absence for a minimum of 24 h of all signs of postoperative acute confusion routinely assessed with the Delirium Observation Screening Scale [21] and an age > 18 years. Patients with diagnosed dementia or other known cognitive impairment were excluded from study participation.

### Sample size calculation

For our primary analysis, there is currently no statistical procedure for a sample size calculation [22]. Thus, we used the intraclass correlation coefficient (ICC) to estimate the sample size. We wanted to obtain an ICC of at least ρ = 0.7 with a 95% confidence interval (CI) from 0.5 to 0.9 and k = 2 ratings per person. In accordance with Bonett [23], this calculation resulted in a total of *n* = 104 participants.

### Measurement process

Six study nurses who were familiar with heart surgery patients conducted the recruitment and data collection.

Patients with an extended stay at the intensive care unit or who had undergone surgery with a heart-lung machine were recruited by a study nurse on day 4 after surgery at the earliest, whereas all other patients were approached on day 1 after surgery at the earliest. If the patient felt unwell or the staff in charge had objections for any health or psychological reason, the measurement was postponed to the next day.

The randomization sequence was computer generated (R Package blockrand, version 1.3) in advance by employing a stratified (by the rater, i.e., study nurses) balanced block randomization. Group allocation was concealed using sequentially numbered opaque sealed envelopes and took place immediately after informed consent was given and baseline data were collected. The participants were randomly assigned to receive either the SEIQoL-DW or the SEIQoL-PF/G as the first measurement in sequence. Immediately after allocation to a sequence group, the patients conducted the respective SEIQoL version either autonomously in the presence of the study nurse (SEIQoL-PF/G) or together with the study nurse (SEIQoL-DW). The study nurses received special training with the SEIQoL-DW instructions [9].

To enable statistical analyses of the agreement, the nominated cues at the first measurement were transferred to the subsequent SEIQoL version.

The other version of the SEIQoL was completed at some point during the following days provided that the patient remained sufficiently stable. We selected a period of 1 to 4 days between the two consecutive measurements to balance between (i) avoiding period effects (which may arise because of potential changes in the mental or health status of the patient), and (ii) eliminating memory effects (which we assumed would bias the results toward better agreement).

Efforts were undertaken to standardize the measurement situation and to minimize the influence of external circumstances. The measurements were conducted in separate rooms. If that was not possible, the study nurse ensured a situation out of sight and out of earshot of other persons. Each patient conducted both measures with the same study nurse.

Data for the study sample description were extracted from the patients’ medical records.

### Statistical analysis

As recommended for metric variables [22, 24], agreement between the two SEIQoL indices was evaluated with Bland-Altman plots, in which the difference between the two sequential measures was plotted against their mean value. Bland-Altman plots allow the interpretation of the extent of differences. Therefore, if limits of agreement (LoAs) are computed, 95% of the sample differences will lie between these limits. Additionally, the 95% CIs of the mean difference and the LoAs were calculated.

To interpret the LoAs, we tried to define a threshold for the determination of their acceptability. Because differences within the scope of half of a standard deviation were considered noticeable in QoL measures [25], and a standard deviation of 20 scale points was applied in a recent study [19], we determined that a difference of 10 scale points was a meaningful threshold. This approach means that to achieve acceptable agreement, the outside limits of the CI of the LoA must be within this range.

Additionally, the ICC of the two indices and its CI was calculated [23] to describe the intraclass correlation.

Bland-Altman plots were also constructed for the cue levels and cue weights, although we did not predefine thresholds.

The effects of instruments or the sequence order as well as the interaction of both of these factors were estimated in a linear mixed model (random intercept only) with corresponding 95% CIs using the R Package nlme, version 3.1–109.

### Quality assurance

Data collection and processing were conducted using pseudonyms. Data entry and monitoring were performed by one researcher (MB) within 3 days of data collection. Any irregularities were clarified immediately with the study staff.

## Results

**Fig. 1:**
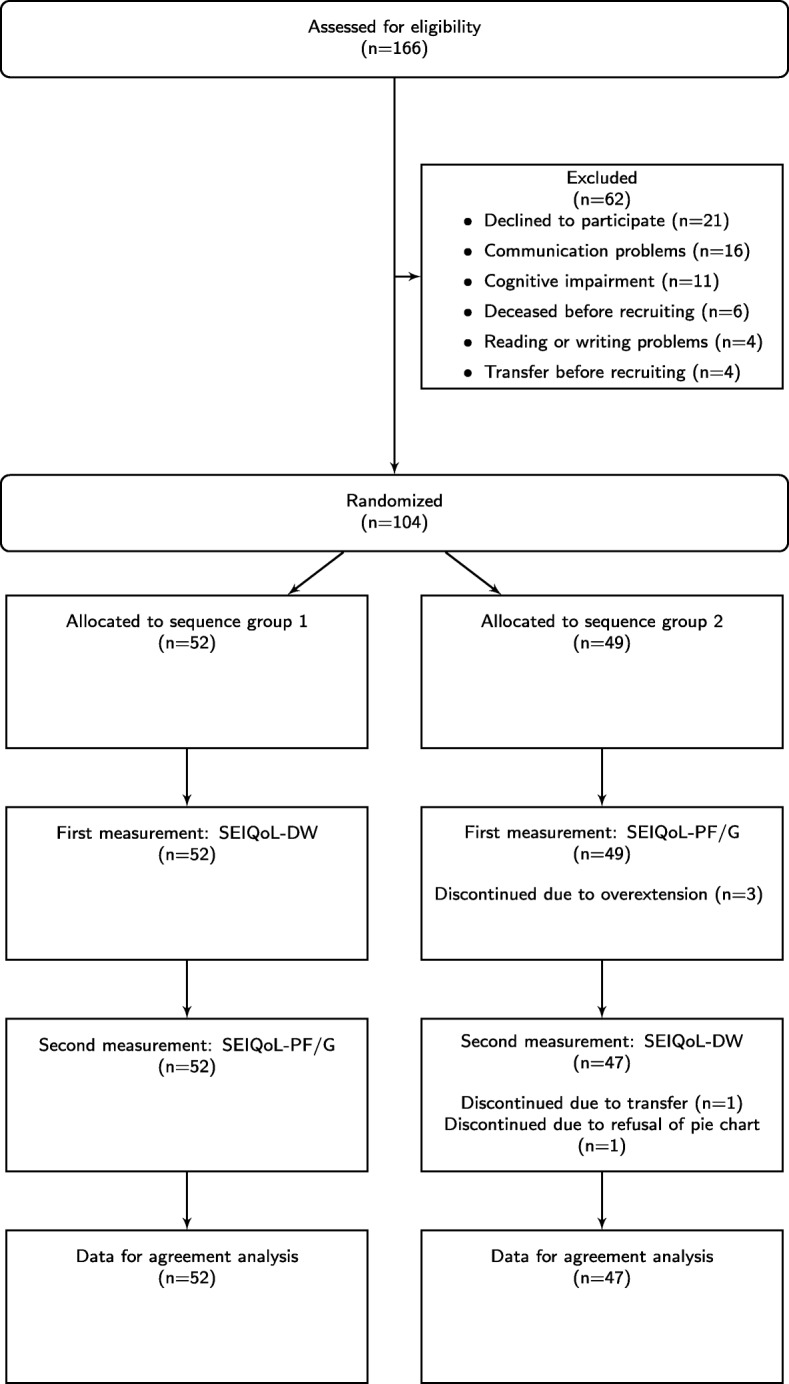
Flow chart of the study participants

During the study period, a total of 166 patients were assessed for eligibility (see Fig. 1), of which we recruited 104 patients. Five patients in sequence group 2 (first measurement: SEIQoL-PF/G) discontinued study participation. Overall, data from *n* = 99 patients were used to estimate the agreement between the instruments.

The average time between the two measurements was 1 day (mean 1.3; SD 0.8).

### Study sample description

Table 1 provides the characteristics of the study participants with both measures grouped by sequence. A slight difference was found only for the type of surgery, because an imbalance existed between the numbers of surgical valve replacements and coronary bypass grafts. However, both procedures are comparable in terms of the postoperative effects on the patient. Sequence group 2 included fewer patients with independent mobility and had a higher rethoracotomy rate due to complications. However, analysis of the subgroup with rethoracotomy showed no deviations in the sociodemographic or clinical data.

**Table 1:** Characteristics of the study participants with both measures

	Sequence group 1 *n* = 52	Sequence group 2 *n* = 47	Total *n* = 99
Age/years, mean (SD)	68.0 (12.0)	65.0 (14.3)	66.6 (13.6)
Gender, n (%)			
Male	40 (76.9)	35 (74.5)	75 (75.8)
Mode of hospital admission, n (%)			
Elective	44 (84.6)	38 (80.9)	82 (82.8)
Emergency	8 (15.4)	9 (19.1)	17 (17.2)
Surgical procedure, n (%)			
Coronary bypass crafting	20 (38.5)	13 (27.7)	33 (33.3)
Surgical valve replacement	13 (25.0)	16 (34.0)	29 (29.3)
Both procedures	7 (13.5)	8 (17.0)	15 (15.2)
Other valve surgery	9 (17.3)	8 (17.0)	17 (17.2)
Other procedure	3 (5.8)	2 (4.3)	5 (5.1)
Length of ICU stay/days, mean (SD)	2.0 (1.5)	2.7 (3.1)	2.3 (2.4)
Acute confusion in the ICU^a^, n (%)	7 (13.5)	7 (14.9)	14 (14.1)
Postoperative complications: rethoracotomy, n (%)	1 (1.9)	5 (10.6)	6 (6.1)
Days after surgery at the first SEIQoL measurement, mean (SD)	5.2 (2.6)	6.1 (4.6)	5.6 (3.7)
Mobility status at the first SEIQoL measurement, n (%)			
Mobility in bed	0	0	0
Mobility up to edge of bed	1 (1.9)	4 (8.5)	5 (5.0)
Mobility to chair	7 (13.5)	9 (19.1)	16 (16.2)
Walking with assistance	11 (21.2)	11 (23.4)	22 (22.2)
Independent mobility	33 (63.5)	23 (48.9)	56 (56.6)

^a^ Routinely assessed with the Delirium Observation Screening Scale

The participants included in this study reflect characteristic features and types of surgical procedures of other heart surgery hospitals in Germany [26].

### Comparison of the SEIQoL-DW and SEIQoL-PF/G for the nominated cues

All patients nominated five cues at the first measurement (sequence group 1: SEIQoL-DW, *n* = 52; sequence group 2: SEIQoL-PF/G, *n* = 49).

Thus, the group performing the SEIQoL-DW first nominated 260 (52 × 5) cues, and the other group named 245 (49 × 5) cues. With few exceptions, no substantial differences were found in the nature and frequency of the cues (see Table 2). Only the cue “religion/spiritual life” was nominated more frequently with the SEIQoL-DW, whereas the cue “travel” was clearly favored with the SEIQoL-PF/G. With both instruments, more than 50% of the patients nominated the areas of life “physical health”, “family”, and “partnership”.

**Table 2:** Comparison of the SEIQoL-DW and SEIQoL-PF/G for the nominated cues collected at the first measurement (SEIQoL-DW: *n* = 52, SEIQoL-PF/G: *n* = 49)

Nominated cues ^a^	Rating level: satisfaction			Relative weight: importance		
	SEIQoL-PF/G, mean (SD)	SEIQoL-DW, mean (SD)	Mean difference (95% CI)	SEIQoL-PF/G, mean (SD)	SEIQoL-DW, mean (SD)	Mean difference (95% CI)
Physical health	63.3 (23.0)	54.95 (32.8)	8.3 (−4.2; 20.8)	21.6 (2.5)	25.7 (11.9)	−4.1 (−7.9; −0.3)
*n* = 80	*n* = 38	*n* = 42				
Family	80.3 (21.4)	83.5 (14.2)	−3.2 (−11.7; 5.3)	22.6 (4.0)	25.5 (8.9)	−3.0 (−6.0; 0.1)
*n* = 78	*n* = 34	*n* = 44				
Partnership	83.5 (17.8)	85.0 (20.5)	−1.5 (− 11.5; 8.5)	21.6 (3.1)	26.7 (10.8)	−5.0 (−9.3; −0.8)
*n* = 59	*n* = 30	*n* = 29				
Friends	71.5 (18.1)	75.0 (16.7)	−3.5 (−13.9; 6.9)	19.7 (1.5)	15.6 (5.9)	4.1 (1.5; 6.6)
*n* = 46	*n* = 22	*n* = 24				
Work/occupation	60.1 (28.5)	61.4 (35.3)	−1.3 (−21.4; 18.9)	18.6 (4.3)	18.7 (10.9)	−0.1 (−5.2; 5.1)
*n* = 41	*n* = 19	*n* = 22				
Nature/garden	70.3 (29.1)	56.1 (31.6)	14.2 (−9.4; 37.7)	18.8 (2.2)	13.2 (6.2)	5.6 (2.0; 9.2)
*n* = 28	*n* = 13	*n* = 15				
Sports	60.7 (29.8)	27.2 (25.9)	33.5 (10.8; 56.1)	19.1 (2.7)	12.3 (6.0)	6.9 (2.7; 11.0)
*n* = 26	*n* = 15	*n* = 11				
Finances	66.6 (14.9)	66.5 (16.2)	0.1 (−19.7; 13.9)	17.9 (2.7)	12.2 (5.8)	5.7 (1.4; 10.1)
*n* = 23	*n* = 13	*n* = 10				
Travel	58.2 (29.7)	60.7 (36.9)	−2.4 (−82.8; 78.0)	17.6 (3.7)	14.3 (5.1)	3.3 (−8.1; 14.7)
*n* = 19	*n* = 16	*n* = 3				
Hobbies	60.7 (19.1)	49.9 (18.4)	10.8 (−8.0; 29.5)	17.8 (2.4)	14.2 (9.3)	3.6 (−3.7; 10.8)
*n* = 18	*n* = 9	*n* = 9				
Food/beverage	60.4 (25.9)	69.9 (23.9)	−9.5 (−37.3; 18.3)	19.1 (3.1)	14.6 (9.1)	4.6 (−4.0; 13.1)
*n* = 15	*n* = 8	*n* = 7				
Leisure activities	69.6 (26.3)	56.1 (34.1)	13.5 (−23.9; 50.9)	18.6 (2.1)	16.1 (8.8)	2.5 (−5.0; 10.0)
*n* = 13	*n* = 5	*n* = 8				
Psychological health	64.8 (30.8)	69.8 (23.5)	−5.0 (−42.0; 32.1)	17.3 (4.5)	19.6 (5.7)	−2.3 (−9.6; 5.0)
*n* = 11	*n* = 6	*n* = 5				
Religion/spiritual life	69.0 (−-)	76.8 (26.6)	−7.8 (−-)	19.0 (−-)	20.1 (6.9)	−1.1 (−-)
*n =* 10	*n* = 1	*n* = 9				

^a^ Cues that were mentioned by at least 10% of all patients

The associated rating of levels was heterogeneous for all cues (see Table 2). The considerable range indicated that some patients tended to rate in the margins of the scale. Only one significant difference was found for the cue “sports”, which was rated higher with the SEIQoL-PF/G than with the SEIQoL-DW.

Considering the associated weighting process, differences between the SEIQoL-DW and SEIQoL-PF/G became obvious, especially given the dispersion of the data; the SEIQoL-DW group showed substantially greater dispersion than the SEIQoL-PF/G group (see Table 2). Although the SEIQoL-DW cues were different in terms of weighting, the SEIQoL-PF/G cues appeared to be nearly equivalent. The cues “physical health” and “partnership” were weighted significantly lower with the SEIQoL-PF/G, whereas the cues “friends”, “nature/garden”, “sports”, and “finances” showed significantly higher values with the SEIQoL-PF/G.

The SEIQoL-DW group had a mean index of 70.0 (SD = 17.1) compared to a mean index of 70.3 (SD = 16.8) for the SEIQoL-PF/G group [mean difference = 0.4 (95% CI, − 6.3 to 7.1)].

### Agreement between the SEIQoL-DW and SEIQoL-PF/G

**Fig. 2:**
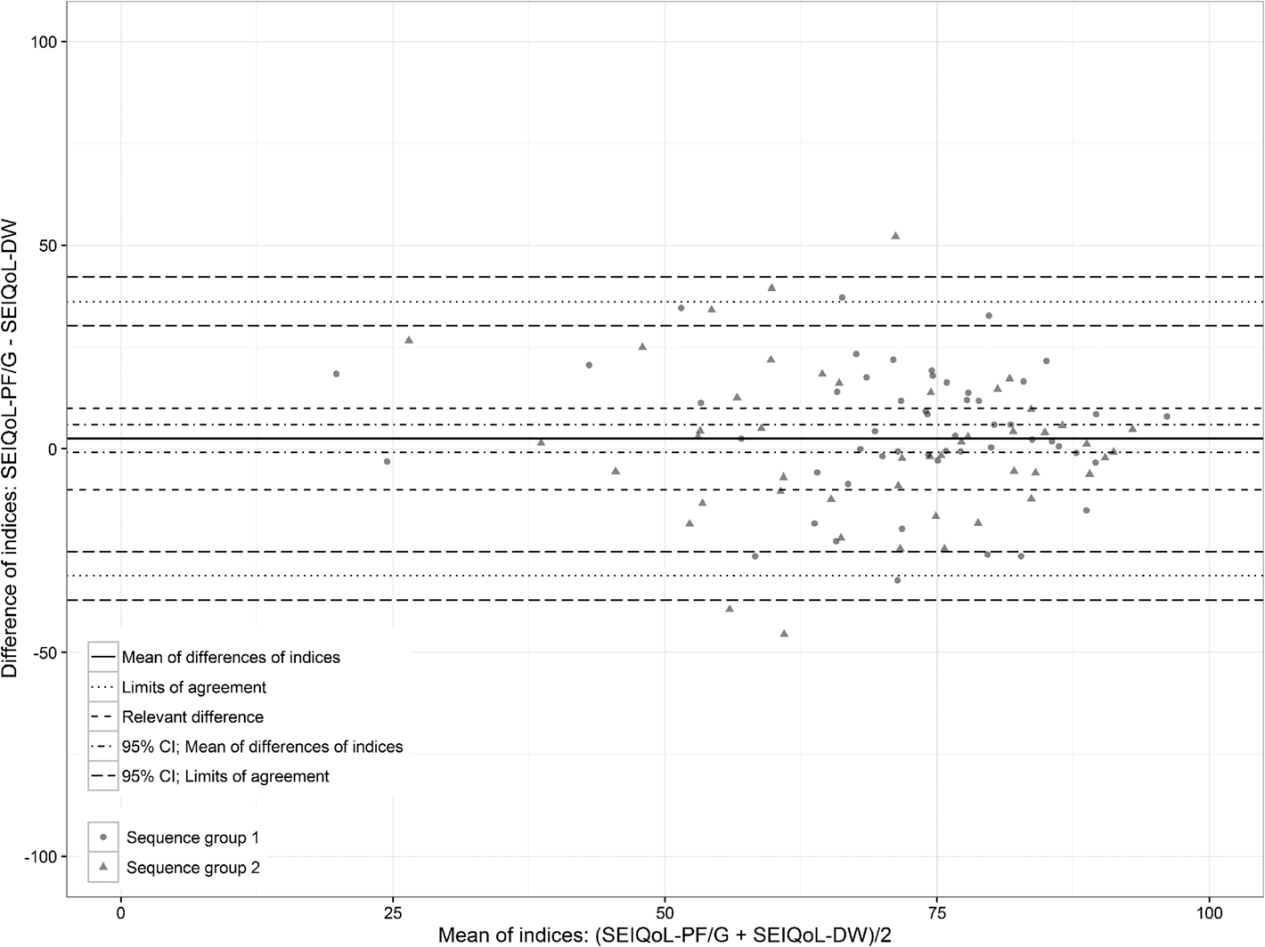
Bland-Altman plot of differences in index measures obtained with the SEIQoL-DW and SEIQoL-PF/G

The agreement between the indices of the two instruments is shown in the Bland-Altman plot (see Fig. 2). The mean of the differences was 2 index points (95% CI, − 1 to 6). The upper LoA showed a difference of 36 index points (95% CI, 30 to 42), and the lower LoA showed a difference of − 31 index points (95% CI, − 37 to − 26). Thus, even in the best case, both LoAs and their CIs were far larger than the predefined tolerable threshold of 10 scale points.

The deviations were also larger if the mean index values ranged between 55 and 75. This result can be mainly explained by the nature of a scale that is calculated as a mean of two scales with inherent scale limits (0 and 100), which results in lower possible differences adjacent to the borders of the mean scale. Given the unambiguous overall result, we did not further analyze any of the outliers.

The ICC that was used to quantify the dependency of both group index values at the two time points indicated a medium correlation: ρ = 0.49 (95% CI, 0.32 to 0.62).

**Fig. 3:**
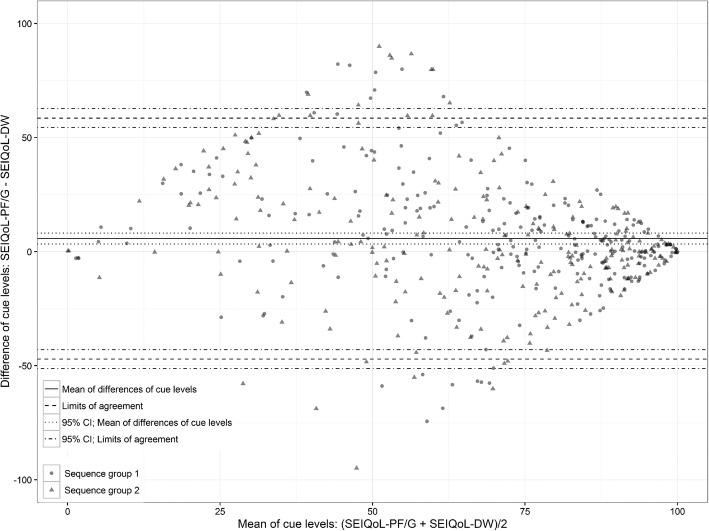
Bland-Altman plot of differences in the cue levels measured with the SEIQoL-DW and SEIQoL-PF/G

The agreement of the cue rating is shown in Fig. 3. The mean difference was 6 scale points (95% CI, 4 to 8), and the LoAs ranged between − 47 (95% CI, − 51 to − 43) and 58 (95% CI, 54 to 63).

The cue rating represents a very large deviation considering a range between 0 and 100. The narrow CIs, which span less than 10 points, indicate a very accurate measure. The agreement is better in the margin areas of the scale than in the middle range, which can be explained by the same mechanism as that applicable for the index. The reduced variability observed here also indicated that the patients often rated their satisfaction in the border areas. This result was similar with both instruments.

**Fig. 4:**
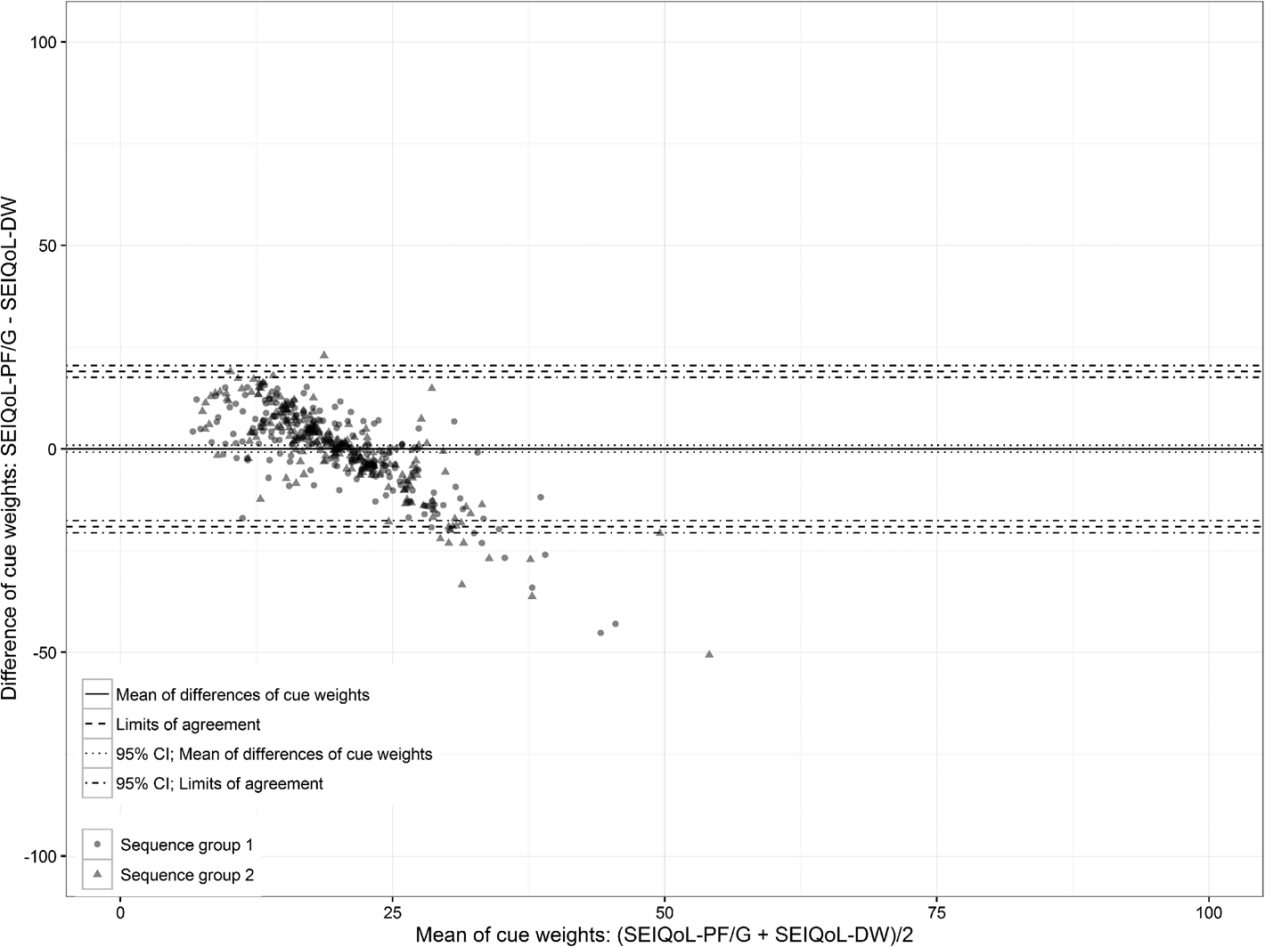
Bland-Altman plot of differences in the weights measured with the SEIQoL-DW and SEIQoL-PF/G

The agreement of the weighting values is shown in Fig. 4. The mean difference was 0 (95% CI, 0 to 1), with LoAs between 19 (95% CI, 17 to 21) and − 19 (95% CI, − 21 to − 17).

Considering that the weight value of 100% is divided between 5 cues with both instruments and that most of the patients weighted their cues below 30%, a nearly 20% deviation is considerable. Moreover, in this plot, a linear relationship that indicates a systematic difference between the instruments is visible. In the weighting range between 5 and 17 scale points, the SEIQoL-DW rated values lower than the SEIQoL-PF/G. Proceeding upwards from 25 scale points, lower weights were given with the SEIQoL-PF/G. This result shows a tendency toward equal weighting of cues in the SEIQoL-PF/G version, while the weighting procedure of the SEIQoL-DW leads to greater variability. An additional analysis of the box plots of the weighted cues of both sequence groups supports this finding (data not shown).

Overall, lower variation exists in the weighting as in the indices or at the cue levels.

No significant instruments or sequence order effects were found (i.e., the results were not time dependent; data not shown).

## Discussion

The aim of our study was to explore the agreement between the SEIQoL-DW and the paper-administered version (SEIQoL-PF/G) in terms of the indices, cue levels, and cue weights.

Our analysis clearly shows a substantial lack of agreement between the SEIQoL-DW and SEIQoL-PF/G. The lack of agreement between the indices became especially evident in our graphic analysis with the Bland-Altman plot. The LoAs in this plot show that the measured index value differences are far higher than the predefined limit of 10 index points.

Due to missing statistical methods for a sample size calculation for this evaluation method, we used the ICC. Because of this provisional nature, sample size was not further adjusted such as an expected loss to follow-up. The ICC of the two indices shows a medium intraclass correlation. Even though this is based on *n* = 5 patients less than in the sample size calculation, the confidence interval, as this is clearly below the original assumptions, implies that it is not likely to have missed a large correlation. However, correlation coefficients suggest a linear relationship of two metric variables, and they should not be used as an agreement assessment [24, 27]. Considering the clear results, we do not expect to achieve better agreement of the two measures in future studies.

This finding is also true for the other Bland-Altman plots in our analyses constructed for cue levels and cue weights. The latter even revealed a systematic difference between the two weighting methods, whereas the other deviations implied random variation of the measured values.

The data of both measures collected for the nominated cues at the first measurement (Table 2) confirm these results.

Both instruments led to similarly nominated cues. By far the most frequently nominated cues were “physical health” and “family”. The cues “family” and “health” were also among the most relevant domains in similar cardiac patient groups in both the SEIQoL-DW [28, 29] and a paper-administered version [12, 18].

The rating of cues (Table 2) was heterogeneous, including one significant difference in the cue “sports”, which had higher ratings in the SEIQoL-PF/G. Interviewer comments revealed that this discrepancy was due to different meanings attached to the cue “sports”, such as “watching sports on TV” and “playing sports”. In accordance with the SEIQoL-DW manual, the interviewers tried to capture the correct meaning when “sports” was rated very high, even if the patient was unable to engage in physical activity a few days after surgery.

For the weighting procedure, the SEIQoL-DW data indicate a relationship between frequent cue nomination and high weighting (Table 2). In contrast, most of the weightings of the SEIQoL-PF/G were nearly equivalent within a narrow range of 20 scale points. Considering the systematic difference found in the Bland-Altman plot, these findings suggest that the SEIQoL-PF/G weighting method is unable to clearly distinguish important from less important cues. Probably, the five separately listed VASs cannot be sufficiently distinguished in terms of their relationships to each other to achieve relative weighting.

However, the mean indices of the SEIQoL-DW and SEIQoL-PF/G group (70.0 vs. 70.3) seem to be unaffected by the deviations in the rating and weighting (shown in Table 2) and show similar values. The SEIQoL-PF/G mean index is considered questionable due to the indifferent weighting method. The SEIQoL-DW mean index is slightly lower (representing a lower quality of life) than that in a comparable setting with cardiac patients [28]. In that study, the index was 81 but referred to patients with minor surgical procedures.

Overall, we can assume that the current SEIQoL-PF/G version is not interchangeable with the SEIQoL-DW as a valid substitute. Nevertheless, a paper-administered measure of individual QoL has great pragmatic importance for use in clinical trials. In our study, the weighting method of the current SEIQoL-PF/G version proved to be problematic. Therefore, the modification of the measure in terms of further development of a differentiating weighting method without interviewer support is desirable. Although the SEIQoL-DW has been explicitly developed for use in clinical practice [7], a more pragmatic version could also be very useful as a tool for care management of patients in clinical practice settings. In particular, identifying areas that are especially important to the individual patient, may support care planning and clinical decision-making [10].

### Strengths and limitations

We assessed agreement between two measures in a randomized crossover design with the concealed allocation of participants to the sequence groups. With a randomized sequence order, it was possible to avoid effects of order of instrument presentation, because we did not know a priori whether the order of completion could have an influence on the results. This procedure corresponds to the recommendations of testing adaptations for agreement with original instruments [30].

Because no validated German translation of the SEIQoL-DW manual existed, it had to be done by the project team. However, these are rather instructions for the study nurses to perform the semi-structured interview than the administration of a questionnaire. The instructions were provided to all study nurses and they received a special training.

The results may be influenced by the time span (mean: 1 day) between the completions of the two versions. Concerning an appropriate period to avoid potential memory effects, we could not find recommendations. Over a 1-week period, however, weaknesses in the test-retest reliability of the SEIQoL-DW in healthy individuals were found [31]. Therefore, to avoid both period effects by health status changes of the patient (from a clinical point of view) and memory effects to the first version, we determined a period of 1 to 4 days. Factors such as emotional states or daytime-dependent variables that could have changed the participants’ judgment could not be assessed. Thus, a clinically founded change in rating or weighting of cues cannot be excluded with certainty. However, we took precautions not to interview patients who felt unwell or had any indication of instability.

Another limitation arises for the interpretation of the ICC in our study. This limitation is associated with the nature of the SEIQoL concept that calculates the score as a multiplicative composite. That means the score is derived from a multiplication of two components: cue rating and cue weighting. The scaling of these variables can have a tremendous impact on correlation with an external measure [32, 33]. A simple linear transformation of the variables can reduce the coefficient or even change the direction of the association [32, 33]. Therefore, the ICC has to be interpreted with caution. However, the ICC is only a secondary indicator in our study; the main analysis is based on the Bland-Altman plots. An approach in future analyses focussing on ICC should apply hierarchical regression modeling [32].

Generally, the studied population was very specific and included only cardiac surgery patients. In that, other populations (e.g. other diseases, healthy population) may lead to different results. However, our study provides valid reference data for acceptable agreement ranges for future research with the SEIQoL-DW. Additionally, the represented main cues embody the perspective of cardiac patients and thus provide a useful adjunct for clinical and research-related issues in this patient group.

## Conclusion

According to our findings, this study is the first to analyze agreement between the original SEIQoL-DW and a version administered without interviewer support. Our data indicate an unacceptable range of agreement between the SEIQoL-DW and the paper-based questionnaire SEIQoL-PF/G for cardiac surgery patients. Thus, use of the current version of the SEIQoL-PF/G as a substitute for the SEIQoL-DW in clinical practice or research is not recommended.

## Supplementary information

**Additional file 1.** SEIQoL-PF/G

## Data Availability

The dataset analyzed during the study is available from the authors upon reasonable request.

## Abbreviations

CI: Confidence interval
ICC: Intraclass correlation coefficient (denoted as ρ, “rho”)
LoA: Limit of agreement
SD: Standard deviation
SEIQoL: Schedule for the Evaluation of Individual Quality of Life
SEIQoL-DW: Schedule for the Evaluation of Individual Quality of Life-Direct Weighting
SEIQoL-PF/G: Schedule for the Evaluation of Individual Quality of Life-Paper Form/German language
QoL: Quality of life
VAS: Visual analogue scale
