# Supplementary material for: Agreement between the Schedule for the Evaluation of Individual Quality of Life-Direct Weighting (SEIQoL-DW) interview and a paper-administered adaption
Source: BMC Med Res Methodol. 2020 Apr 10;20:80. doi: 10.1186/s12874-020-00961-9 (PMC7149856; doi:10.1186/s12874-020-00961-9)
Supplement: Supplementary file 1 — Additional file 1. SEIQoL-PF/G [file 12874_2020_961_MOESM1_ESM.pdf]

Additional file 1: SEIQoL-PF/G (adapted from Becker et al. 2009) English

The following questionnaire will help assess **your quality of life**. The questions allow you to assign weights to different aspects that are important for your life and to determine your individual quality of life.

❶ In the first step, we ask you **to name the 5 most important areas of your life** at present … the things that determine the quality of your life.
❷ In the second step, we ask you to rate **how satisfied you are in each of these 5 areas.** (Please mark with a vertical line.)
❸ In the final step, we want to know **how important each of these 5 areas is to you**. (Please mark with a vertical line.)

<u>Examples of areas of life:</u> partnership, work, food/beverage, family, finances, friends, hobbies, physical health, nature/garden, psychological health, travel, religion/spiritual life, sports, leisure activities…

The examples should encourage you to elicit your own areas of life. However, you are also welcome to use one or more of these areas of life.

❶ *Life area*	❷ Strength of **satisfaction**<br>(Satisfaction increases with the rise.)	❸ Strength of **importance**<br>(Importance increases with the rise.)
1)		
2)		
3)		
4)		
5)		

Im Folgenden geht es um die Beurteilung **Ihrer eigenen Lebensqualität**. Die Fragen ermöglichen es, die einzelnen Bereiche, die für Ihre persönliche Lebensqualität wichtig sind, zu gewichten und Ihre individuelle Lebensqualität zu ermitteln.

- ❶ Sie werden zunächst gebeten, **5 wichtige Bereiche aus Ihrem Leben zu nennen**, die einen Einfluss auf Ihre Lebensqualität haben.
- ❷ Im zweiten Schritt bitten wir Sie, uns zu sagen, **wie zufrieden Sie in jedem dieser 5 Bereiche sind**. (Bitte mit senkrechtem Strich markieren.)
- ❸ Im letzten Schritt möchten wir wissen, **wie wichtig Ihnen jeder dieser 5 Bereiche ist**. (Bitte mit senkrechtem Strich markieren.)

Beispiele für Lebensbereiche: Beruf/Beschäftigung, Ehe/Partnerschaft, Essen und Trinken, Familie, Finanzen, Freunde/sozialer Bereich, Hobbys/Freizeitgestaltung, körperliche Gesundheit, Natur/Garten, psychologische Gesundheit, Reisen, Religion/Spiritualität, Sport, Unterhaltung…
Die Beispiele sollen Sie dazu ermuntern, eigene Lebensbereiche zu nennen. Sie können aber auch gerne Lebensbereiche übernehmen.

❶ *Lebensbereich*	❷ Stärke der **Zufriedenheit** (Die Zufriedenheit nimmt mit der Steigung zu.)	❸ Stärke der **Wichtigkeit** (Die Wichtigkeit nimmt mit der Steigung zu.)
1)		
2)		
3)		
4)		
5)		
